# Fc-effector functional antibody assays for SARS-CoV-2 variants of concern

**DOI:** 10.3389/fimmu.2025.1571835

**Published:** 2025-05-20

**Authors:** Xuemin Chen, Grace Li, Caroline Ciric, Theda Gibson, Larry J. Anderson, Christina A. Rostad

**Affiliations:** ^1^ Division of Infectious Diseases, Department of Pediatrics, Emory University School of Medicine, Atlanta, GA, United States; ^2^ Department of Pediatrics, Children’s Healthcare of Atlanta, Atlanta, GA, United States

**Keywords:** ADCC, ADNP, ADCP, non-neutralizing antibodies, cytotoxicity, phagocytosis, complement deposition

## Abstract

**Background:**

The Fc regions of antibodies mediate important effector cell functions such as antibody-dependent cellular cytotoxicity (ADCC), antibody-dependent cellular phagocytosis (ADCP), antibody-dependent neutrophil phagocytosis (ADNP), and complement-dependent cytotoxicity (CDC). These functions enhance immune defense and infected cell clearance. This study evaluated the effect COVID-19 XBB.1.5 booster vaccination on Fc-effector antibody responses to SARS-CoV-2.

**Methods:**

We developed four assays to evaluate the Fc-effector functions of SARS-CoV-2 antibodies. ADCC and CDC assays utilized stably transfected luciferase-based target cell lines expressing SARS-CoV-2 spike variants (Ancestral, Wu-1 Omicron XBB.1.5, and EG.5) to measure antibody-mediated lysis by effector cells. ADCP and ADNP were assessed by flow cytometry to measure phagocytosis of fluorescently labeled virus-like particles that display SARS-CoV-2 variant spike proteins. Serum samples from 20 healthy adult volunteers pre- and post-monovalent XBB.1.5 COVID-19 vaccine were analyzed for pseudovirus neutralizing and Fc-effector antibodies.

**Results:**

Prior to administration of the COVID-19 XBB.1.5 booster vaccination, cross-neutralizing antibodies against XBB.1.5 and EG.5 variants were minimally detectable, while cross-functional Fc-effector antibodies were present at higher baseline levels. The COVID-19 XBB.1.5 booster vaccination significantly boosted both neutralizing and Fc-effector antibodies in magnitude and breadth. The greatest increase in neutralizing antibodies was against the XBB.1.5 strain, while Fc-effector functional antibodies had similar fold-increases in antibody titers against the breadth of SARS-CoV-2 variants tested. Neutralizing and Fc-effector antibodies were most highly correlated at baseline (prior to booster vaccination) but were less correlated post-vaccination, consistent with differential boosting of neutralizing vs Fc-effector antibodies by the monovalent vaccine.

**Conclusion:**

The COVID-19 XBB.1.5 booster vaccination significantly improved the magnitude, breadth, and quality of antibody responses to SARS-CoV-2. Combining Fc-mediated functional and neutralizing antibody assays provides a more comprehensive model for understanding vaccine-induced immunity and optimizing vaccination strategies.

## 1 Introduction

Fc-effector antibodies are an important component of the humoral immune response to viruses including the severe acute respiratory syndrome coronavirus 2 (SARS-CoV-2), which emerged in 2019. As SARS-CoV-2 continues to evolve, including measures of antibody-mediated Fc-effector functions in immune assessments is especially important for understanding vaccine-induced immunity to SARS-CoV-2 variants. A more complete picture of vaccine-induced immunity to SARS-CoV-2 variants may help optimize vaccine design (1–3). Fc-effector functions, including antibody-dependent cellular cytotoxicity (ADCC), complement-dependent cytotoxicity (CDC), antibody-dependent cellular phagocytosis (ADCP), and antibody-dependent neutrophil phagocytosis (ADNP), play an important role in the immune response by facilitating the clearance of infected cells and enhancing the overall immune defense. Recent studies have shown that COVID-19 vaccination can significantly increase the quality and magnitude of Fc-effector antibody responses (4–6). Vaccination generally leads to stronger Fc-mediated immune responses, characterized by increased binding affinity of antibodies to Fc receptors on immune cells such as natural killer (NK) cells and macrophages (4, 7–9). This enhanced binding translates to more effective engagement and activation of these effector cells, resulting in improved clearance of the virus.

However, the relationship between COVID-19 vaccination and Fc-effector antibody responses is complex and may vary depending on the individual and the specific variant of concern. SARS-CoV-2 variants, including Delta and Omicron, have mutations in the spike protein that have been shown to decrease binding of antibodies induced by prior infection or vaccination (10–12). These changes can impede the ability of antibodies to mediate neutralization and Fc-effector functions, thereby affecting the overall effectiveness of the immune response (11, 13–17).

Studies comparing different COVID-19 vaccination regimens have highlighted the importance of booster doses in maintaining robust Fc-effector antibody functions (10, 18). Booster doses updated to current circulating strains not only enhance the quantity of antibodies but also improve their functional quality, which is likely particularly important for variants with neutralizing escape mutations. For example, booster vaccinations have been shown to restore and even enhance Fc-effector functions against variants like Beta and Gamma, which partially evade neutralizing antibodies elicited by the initial vaccination series (19–21). This is likely in part because booster vaccination elicits both neutralizing and non-neutralizing antibodies to SARS-CoV-2 variants with Fc-effector capacity.

Overall, understanding Fc-effector antibody responses to COVID-19 vaccination may help optimize vaccine design and guide booster recommendations as SARS-CoV-2 variants emerge. This study describes the development of a panel of Fc-effector antibody assays against SARS-CoV-2 variant spikes and the effect of the COVID-19 XBB.1.5 booster vaccination on functional antibody responses to recent and past SARS-CoV-2 variants in a cohort of healthy adults.

## 2 Results

### 2.1 Study cohort

We enrolled 20 adults with median age 43 years (IQR 32, 56), 70% female, 70% White, 10% Black, 10% Asian, 10% Multiple races, and 15% Hispanic ethnicity (**Table 1**). Prior to enrollment, 85% of participants had been previously diagnosed with COVID-19, and all participants had received COVID-19 vaccinations (mean 4.0 vaccinations, SD 0.76). We collected sera at baseline and one-month post-XBB.1.5 COVID-19 vaccination. Nine (45%) participants received the Moderna XBB.1.5 booster, and 11 (55%) received the Pfizer formulation.

**Table 1:** Study cohort and baseline SARS-CoV-2 status.

Sex	Race	Ethnicity	Past COVID infections	Past COVID vaccines	Past COVID vaccine brands	XBB1.5 Booster Brand
Female	White	Non-Hispanic	2	5	M,M,M,M,M	Moderna
Male	White	Non-Hispanic	2	4	P,P,P,P	Pfizer
Female	White	Hispanic	1	3	P,P,P	Pfizer
Female	Black	Non-Hispanic	1	5	P,P,P,P,M	Pfizer
Female	White	Non-Hispanic	2	4	M,M,M,M	Pfizer
Male	Asian	Non-Hispanic	1	4	M,M,P,P	Moderna
Female	White	Non-Hispanic	1	4	P,P,P,P	Pfizer
Male	White	Non-Hispanic	0	4	M,M,P,P	Moderna
Female	White	Non-Hispanic	0	4	P,P,P,M	Pfizer
Male	White	Hispanic	2	4	P,P,P,P	Pfizer
Male	White	Non-Hispanic	1	4	P,P,M,P	Pfizer
Female	White	Non-Hispanic	2	4	M,M,M,M	Moderna
Female	White	Non-Hispanic	1	5	M,M,M,P,P	Pfizer
Female	White	Non-Hispanic	1	2	J,P	Moderna
Female	Multiple races	Non-Hispanic	1	4	M,M,M,M	Moderna
Female	Asian	Non-Hispanic	1	4	P,P,P,P	Pfizer
Female	White	Non-Hispanic	1	5	M,M,M,P,M	Moderna
Male	White	Hispanic	1	4	P,P,P,P	Moderna
Female	Black	Non-Hispanic	0	3	J,J,M	Pfizer
Female	Multiple races	Non-Hispanic	2	5	M,M,M,M,M	Moderna

J, Janssen; M, Moderna; P, Pfizer. Vaccine brands are in order of receipt.

### 2.2 Development of assays to measure functional Fc effector antibodies

#### 2.2.1 Assays to measure antibody-dependent cellular phagocytosis and neutrophil phagocytosis

**Figure 1:**
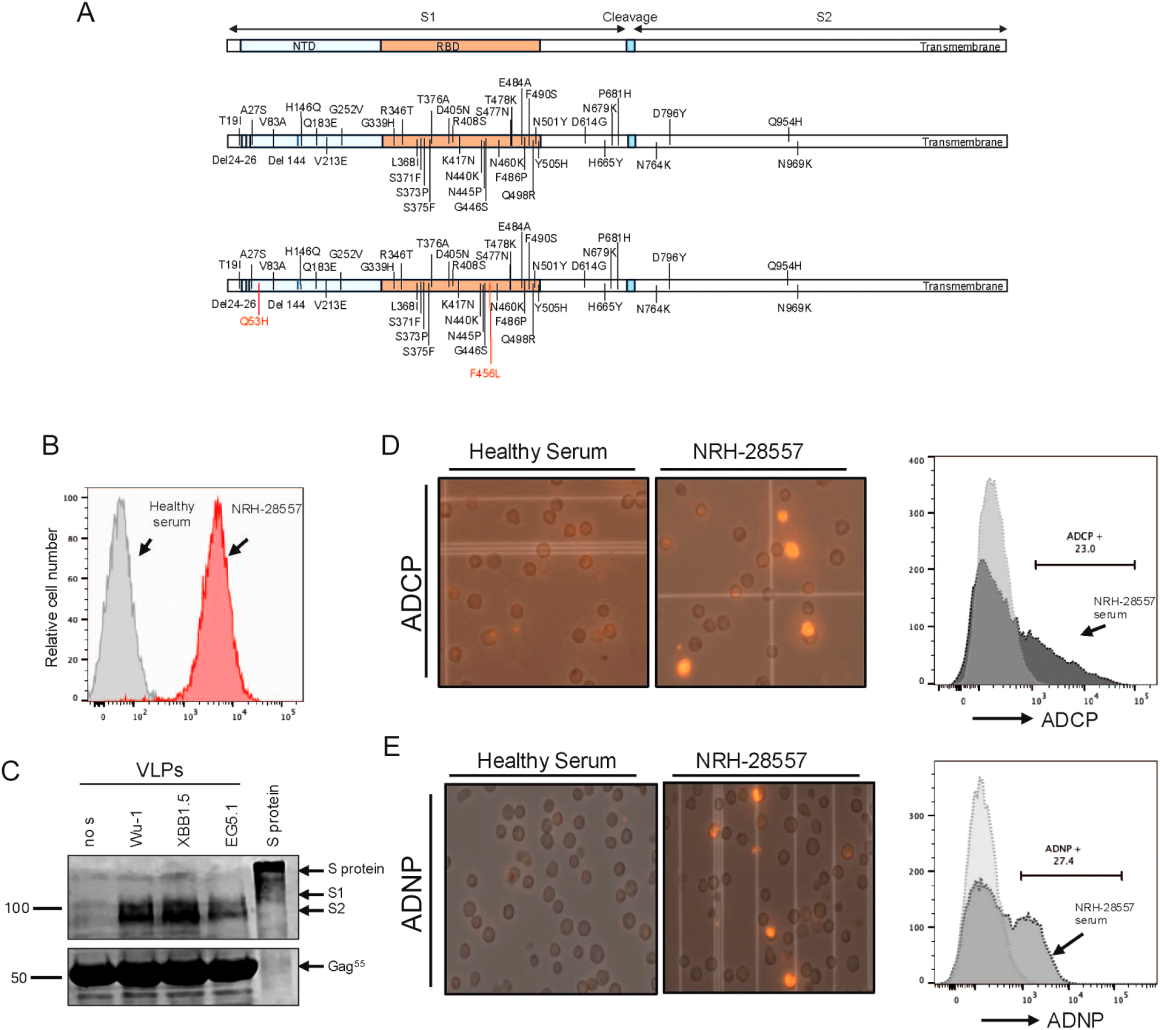
Characterization and recognition of HIV gag virus-like particles functionated with SARS-Cov-2 spike protein by SARS-CoV-2 polyclonal serum. **(A)** Schematic representation of the amino acid change in the spike protein of XBB.1.5 and EG.5 compared to ancestral variant Wu-1. **(B)** Flow cytometry analysis of G protein expression on the VLP production cell surface. **(C)** Western blot analysis of purified VLPs using COVID-19 vaccinated serum as primary antibody for detection of spike protein. Full lengths protein was used as control. **(D)** An example of flowcytometry analysis of Antibody-Dependent Cellular Phagocytosis (ADCP) activity (left). Microscopy images of THP1 cells demonstrate phagocytotic activity induced by COVID 19 vaccinated serum compares to healthy serum (right). **(E)** An example of flow cytometry analysis of Antibody-Dependent Neutrophile Phagocytosis (ADNP) activity (left). Microscopy images of HL60 cells demonstrate phagocytotic activity induced by COVID 19 vaccinated serum compares to healthy serum (right).

To develop ADCP and ADNP assays, we first generated HIV-gag virus-like particles (VLPs) that incorporated the SARS-CoV-2 variant spike proteins, Wu-1, XBB.1.5, or EG.5 (**Figure 1A**). To do this, we developed stable, inducible cell lines using T-REx 293 cells which allowed for consistent, reproducible spike protein expression and stable spike-to-Gag protein ratio upon doxycycline induction. Spike glycoprotein expression on the VLP production cell surface was confirmed by flow cytometry analysis (**Figure 1B**) and total expression by Western blot (**Figure 1C**) using the polyclonal anti-serum from COVID-19 vaccinated individuals (BEI NRH 28557) as the primary antibody.

We then fluorescently labeled the production cell line with a lipophilic fluorescent dye DiD before inducing VLP production. DiD is a stable carbocyanine dye that integrates into cell membranes, providing uniform staining without affecting cellular functions. Because of its structural similarity to phospholipids, DiD can persist in cells for extended periods without disrupting cellular processes, making it ideal for labeling living cells.

We then optimized assays to measure antibody-mediated phagocytosis of the DiD fluorescently labeled VLPs by THP-1 (ADCP) and differentiated HL-60 (ADNP) effector cell lines. VLPs were incubated with serially diluted serum and then cultured with the effector cells. Phagocytosis by ADCP (**Figure 1D**) and ADNP (**Figure 1E**) was measured using fluorescent microscopy and flow cytometry. The representative image taken with an EVOS microscope shows phagocytosis of fluorescent VLPs by THP-1 and HL-60 cells, which occurred when fluorescent VLPs incubated with COVID+ reference serum (BEI Resources). In contrast, fluorescent VLPs incubated with healthy control serum show little or no phagocytosis by either effector cell line. The effector cells were gated and analyzed for the presence of DiD fluorescence at a spectrum of 670 nm, marking phagocytic cells that were considered positive. As shown in **Figures 1D, E**, the BEI reference serum induced 23% positivity in ADCP and 27% in ADNP at a dilution of 1:20. Conversely, background levels were observed in samples treated with VLPs alone or with the serum from healthy controls in both assays. The background signal induced by VLPs alone was subtracted from the tested samples when calculating the percentage of cells with phagocytosis.

**Figure 2:**
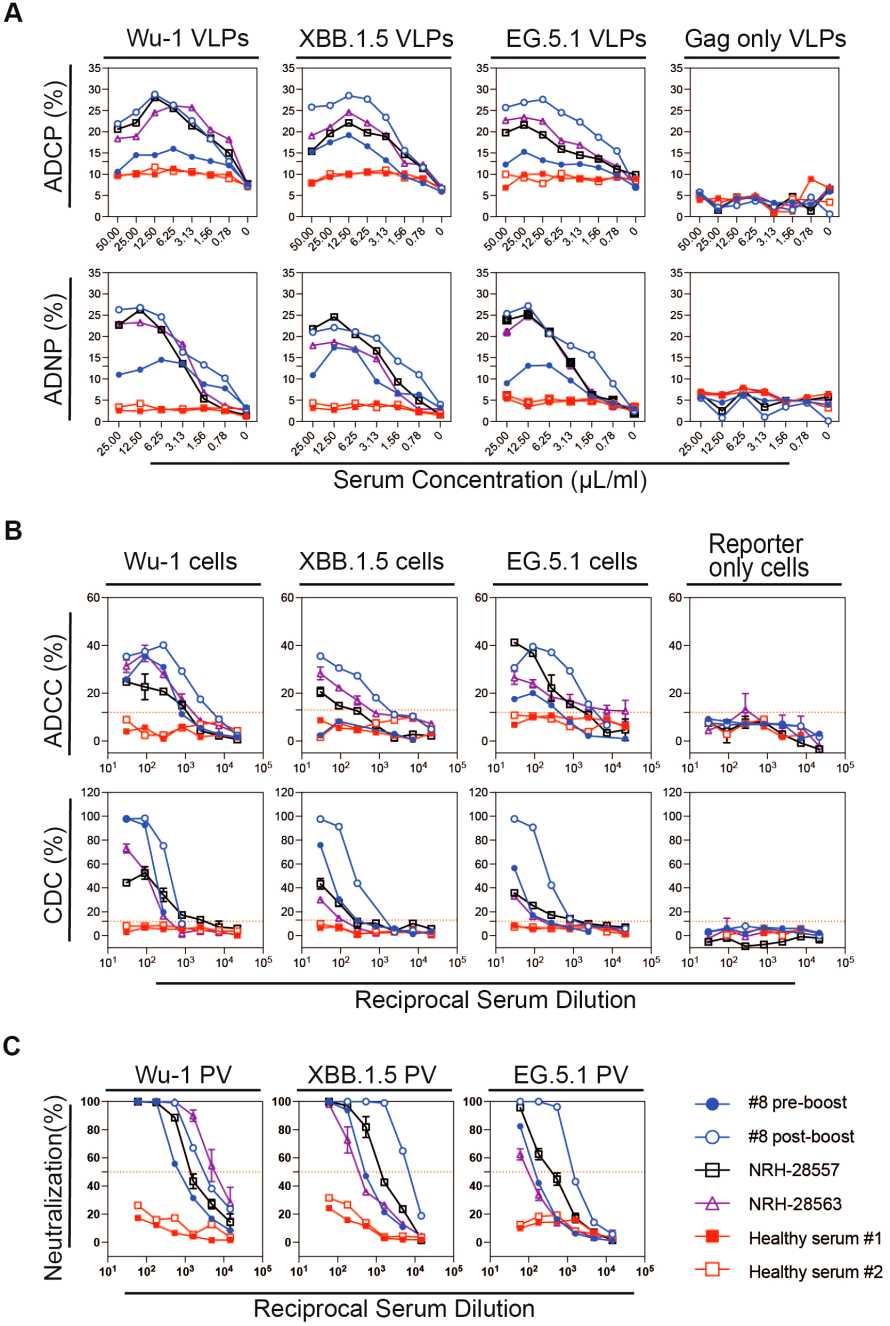
Representative data of Fc effector and pseudovirus neutralizing antibody responses in serially diluted sera from a participant (#8) with pre- and post-XBB1.5 booster serum samples, from two donors collected prior to the COVID-19 pandemic with an unknown history of coronavirus infection (pre-pandemic) noted as healthy serum #1 and #2, and from two reference sera samples obtained from BEI Resources (NRH-28557 and NRH-28563) for **(A)** ADCP and ADNP, **(B)** ADCC and CDC, and **(C)** pseudovirus neutralizing antibody responses against SARS-CoV-2 variant spike proteins (Ancestral Wu-1, Omicron XBB.1.5, and EG.5). The red dashed line indicates 50% reduction in viral infection in neutralization assay, and cut-off was calculated from 12 serum samples collected prior to the COVID-19 pandemic in ADCC and CDC.

To optimize the ratio of VLPs to serum, we normalized the VLP quantity based on HIV Gag p24 protein levels and incubated the VLPs with serially diluted serum. A serum concentration–response curve is shown in **Figure 2A**, where VLPs were incubated with progressively lower serum concentrations. At higher serum concentrations, we observed a prozone effect, characterized by reduced phagocytic activity after reaching a peak. Maximum phagocytic activity occurred at a serum concentration of 12.5 µL/mL, after which a decline in activity was noted. This concentration-dependent effect was observed in both the ADCP and ADNP assays, with similar response curves. These findings suggested that excessive antibody binding may interfere with optimal phagocytosis. Therefore, based on this experiment, a serum concentration of 12.5 µL/mL was determined to be optimal for observing maximum phagocytic activity.

To confirm the reproducibility of the assays, we ran the same samples using the same source of THP-1 cells and differentiated HL-60 cells across two different days, comparing the consistency of the results. The reference sera consistently mediated high phagocytosis activity, while the healthy control sera mediated minimal to no phagocytosis (**Supplementary Figures S1**, **S2**).

#### 2.2.2 Assays to measure antibody-dependent cellular cytotoxicity and complement-dependent cytotoxicity

To measure ADCC and CDC, we utilized target cell lines with tetracycline-inducible expression of SARS-CoV-2 spike proteins and reporter proteins luciferase and eGFP as previously described (26). In this study, we developed two new target cell lines expressing SARS-CoV-2 variant spikes, XBB.1.5 and EG.5, in addition to the previously generated target cell line expressing SARS-CoV-2 ancestral (Wuhan-1) spike. We measured the inducible surface expression of SARS-CoV-2 spike protein for the three target cell lines using COVID-positive BEI reference serum NRH-28557 and found them to have similar surface spike protein expression by flow cytometry as measured by percentage positive cells and median fluorescence intensity (MFI) (**Supplementary Figure S3**). We additionally characterized the target cells using sera collected from study participants pre- and post-COVID-19 vaccination, and 12 healthy controls. The sera were tested in a series of three-fold dilutions, starting at a 1:30 dilution, in a 96-well plate format. We assessed the ADCC activity of these sera by using target cells with and without spike protein expression, and effector cells with NK CD16(+) receptors. As shown in **Figure 2B**, all four vaccinated serum samples induced lysis of the S protein-expressing target cells by NK CD16(+) effector cells. In contrast, sera from the pre-COVID-19 pandemic samples elicited only background levels of target cell lysis. Cut-off values for end-point titer calculation were determined based on the cell lysis induced by the healthy control sera: 12% for Wu-I and EG.5, and 13% for XBB.1.5. Control target cells, which lacked spike protein expression, did not undergo significant lysis in the presence of BEI reference sera and NK CD16(+) effector cells (data not shown).

In addition to antibody-mediated cytotoxicity, we evaluated CDC, which occurs when antibodies engage their target and trigger complement C1q binding to the Fc region, leading to the target cell lysis. We developed and optimized a CDC assay using the same target cell lines as the ADCC assay. To determine the optimal complement concentration, we tested 1/20 and 1/40 dilutions of guinea pig complement in the presence of BEI reference sera, participant sera post-vaccination, and healthy control sera over a 28-hour incubation period (**Supplementary Figure S4**). Higher complement concentrations increased target cell lysis, while spontaneous killing remained under 13% with healthy control sera. Notably, increasing the complement concentration from 1/40 to 1/20 did not increase spontaneous killing, and thus, the 1/20 complement concentration was selected for further experiments. We also examined the effect of incubation time on CDC activity at incubation times of 6, 12, and 28 hours. The optimal CDC response was observed after 28 hours (**Supplementary Figure S5**).

With this optimized assay, we found that antibodies from BEI reference sera and participant sera post-vaccination, in combination with guinea pig complement, effectively induced CDC, resulting in target cells lysis. In contrast, serum samples from healthy controls did not induce lysis in any of the three target cell lines. As expected, no CDC activity was detected in the control cell line lacking spike expression, whether tested with the positive or negative sera (**Figure 2B**). These findings demonstrate that antibodies from vaccinated individuals can effectively activate the complement cascade, leading to cell lysis via CDC. The pseudovirus neutralizing antibody assay curves for the corresponding sera are shown in **Figure 2C**. Additionally, we performed ROC analyses to establish the optimal thresholds for the ADCC and CDC using serum samples from 12 healthy controls, determining cut-off values for the Wu-1 and the Omicron subvariants (XBB.1.5 and EG.5) (**Supplementary Figure S6**, **Supplementary Table S1**) and evaluated the assay reproducibility (**Supplementary Figure S7**).

### 2.3 Fc-effector antibody responses to SARS-CoV-2 spike variants following COVID-19 XBB.1.5 booster vaccination

**Figure 3:**
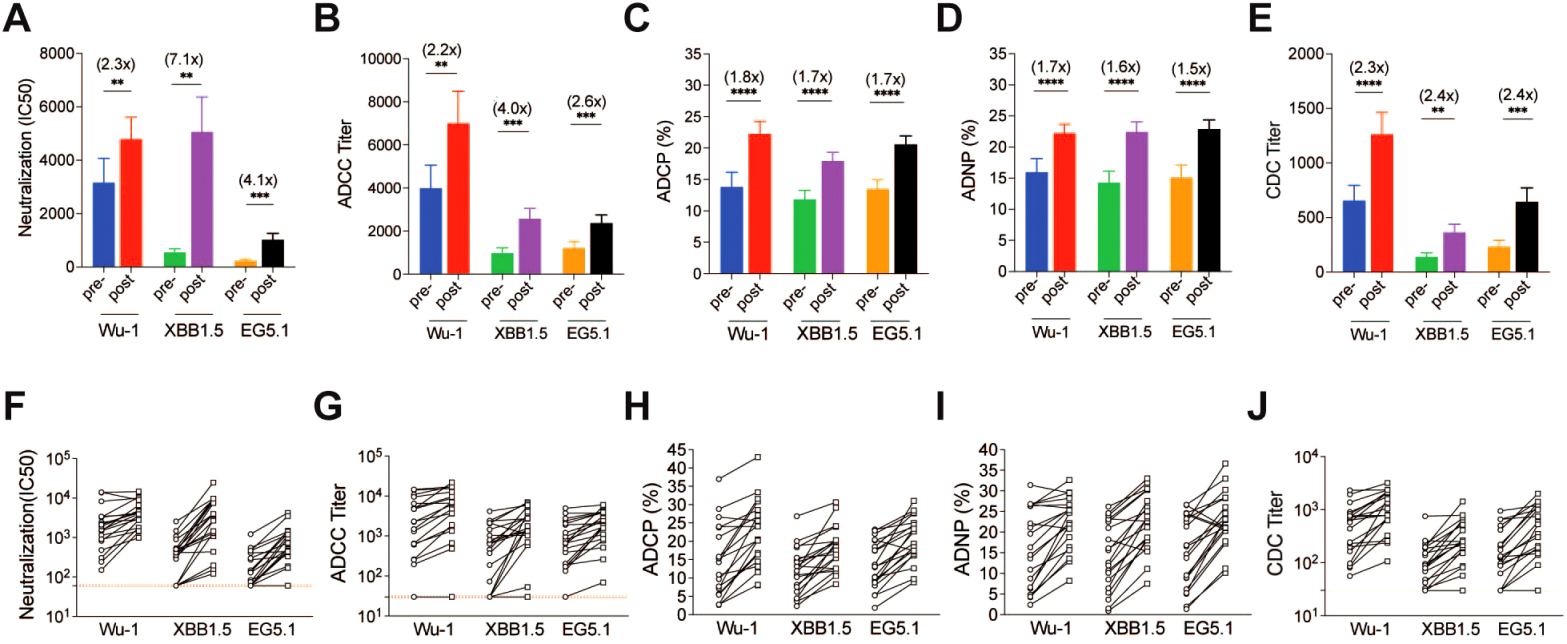
SARS-CoV-2-specific antibody responses against Wu-1, XBB.1.5, and EG.5 in adults pre- and post-COVID-19 XBB.1.5 booster vaccination. **(A)** SARS-CoV-2 S protein–specific neutralization IC50 titers were determined using a pseudotyped virus neutralization assay. **(B)** Functional antibody-dependent cell-mediated cytotoxicity (ADCC) responses were evaluated, measuring the capacity of antibodies to mediate the destruction of target cells by natural killer cells. **(C)** Antibody-dependent cellular phagocytosis (ADCP) activity and **(D)** antibody-dependent neutrophil phagocytosis (ADNP) activity were assessed, indicating the ability of antibodies to promote phagocytosis of fluorescent virus-like particles by phagocytes and neutrophils. **(E)** Complement-dependent cytotoxicity (CDC) responses were measured, reflecting the ability of antibodies to induce complement-mediated lysis of target cells. Statistical comparisons were made using paired T-tests (**P < 0.01, ***P < 0.001, ****P < 0.0001). The fold changes between pre- and post-COVID-19 XBB.1.5 booster vaccination are shown in brackets. F to J show the individual spike-specific neutralization IC50 titers **(F)**, ADCC end-point titers **(G)**, ADCP phagocytosis percentage **(H)**, ADNP phagocytosis percentage **(I)** and CDC end-point titers **(J)** against Wu-1, XBB.1.5 and EG.5 pre- and post-COVID-19 XBB.1.5 booster vaccination.

After development of the panel of assays, we then measured pseudovirus neutralization and Fc-effector antibody responses to the SARS-CoV-2 spike variants pre- and post-COVID-19 XBB.1.5 booster vaccination for the 20 enrolled study participants. Prior to administration of the COVID-19 XBB.1.5 booster vaccination, cross-neutralizing antibodies against XBB.1.5 and EG.5 variants were minimally detectable, while cross-functional Fc-effector antibodies were present at higher baseline levels (**Figure 3**). The COVID-19 XBB.1.5 booster vaccination significantly boosted both neutralizing and Fc-effector antibodies in magnitude and breadth. The greatest increase in pseudovirus neutralizing antibodies was against the XBB.1.5 strain (7.1-fold), followed by EG.5 (4.1-fold), and Wu-1 (2.3-fold). Similarly, the greatest increase in ADCC antibody responses was against the XBB.1.5 strain (4.0-fold), followed by EG.5 (2.6-fold) and Wu-1 (2.2-fold). Interestingly, ADCP, ADNP, and CDC antibodies had similar fold-increases in antibody titers against the breadth of SARS-CoV-2 variants tested when compared to baseline. ADCP antibodies increased for XBB.1.5 (1.7-fold), EG.5 (1.7-fold), and Wu-1 (1.8-fold); while ADNP antibodies increased to XBB.1.5 (1.6-fold), EG.5 (1.6-fold), and Wu-1 (1.7-fold). CDC antibodies increased to XBB.1.5 (2.4-fold), EG.5 (2.4-fold), and Wu-1 (2.3-fold).

### 2.4 Correlation between neutralization and Fc function

**Figure 4:**
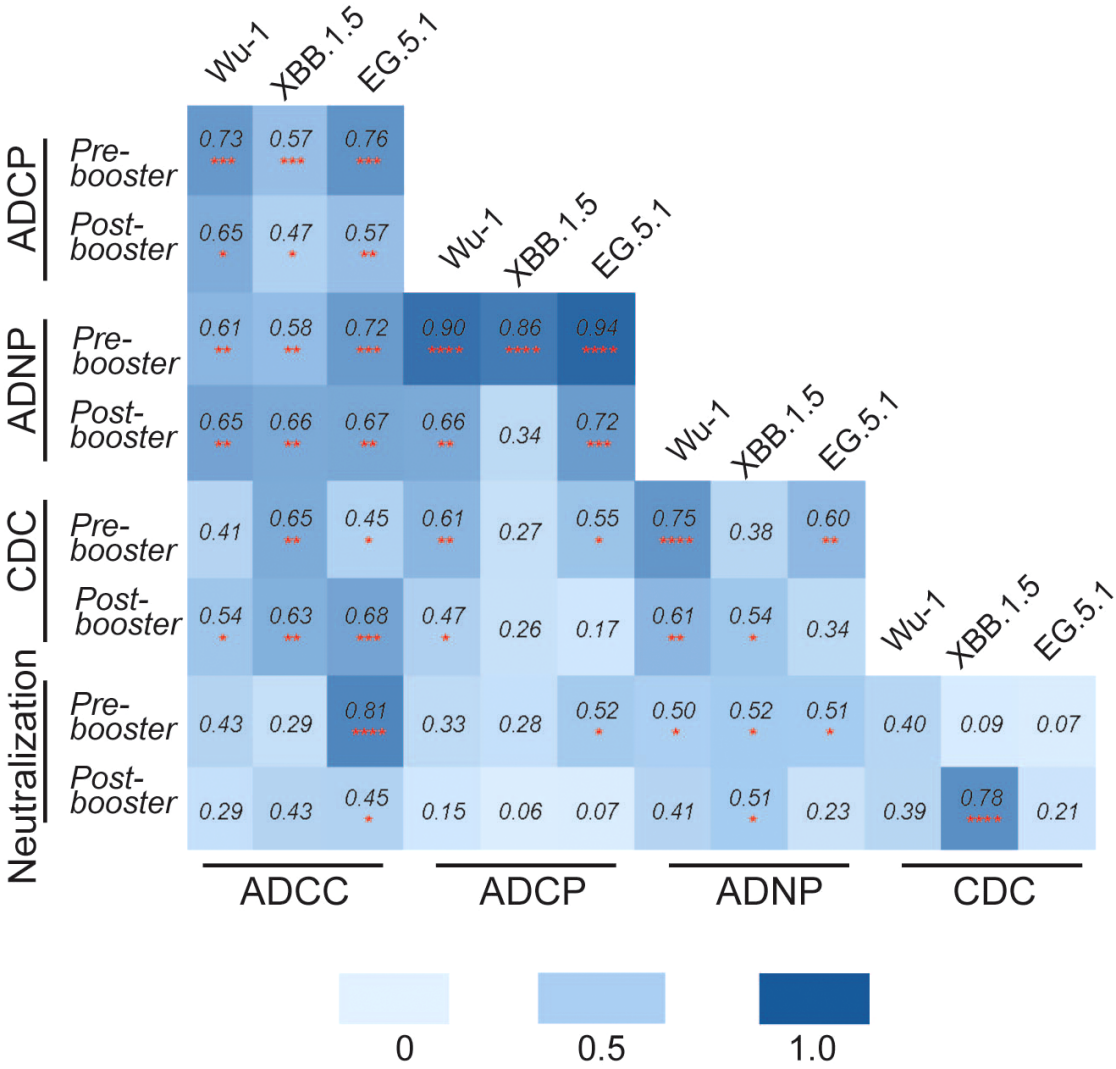
Pearson’s correlation matrix analysis was performed to analyze the correlation between different functional antibody responses against SARS-CoV-2 variants Wu-1, XBB.1.5, and EG.5 pre- and post-COVID-19 XBB.1.5 booster vaccination. The colored squares show the correlation coefficient between two variables, with darker blue color representing a stronger correlation. The red asterisks indicate the level of statistical significance (*p < 0.05, **p < 0.01, ***p < 0.001, ****p<0.0001).

We then measured Pearson’s correlation between neutralizing and Fc-effector antibody responses to the SARS-CoV-2 variants (**Figure 4**). Analysis revealed a variable but generally positive correlation between neutralization IC50 values and Fc-effector antibody titers. We found that neutralizing and Fc-effector antibodies were most highly correlated at baseline (prior to booster vaccination) but were less correlated post-vaccination, consistent with differential boosting of neutralizing vs Fc-effector antibodies to SARS-CoV-2 spike by the monovalent vaccine.

**Figure 5:**
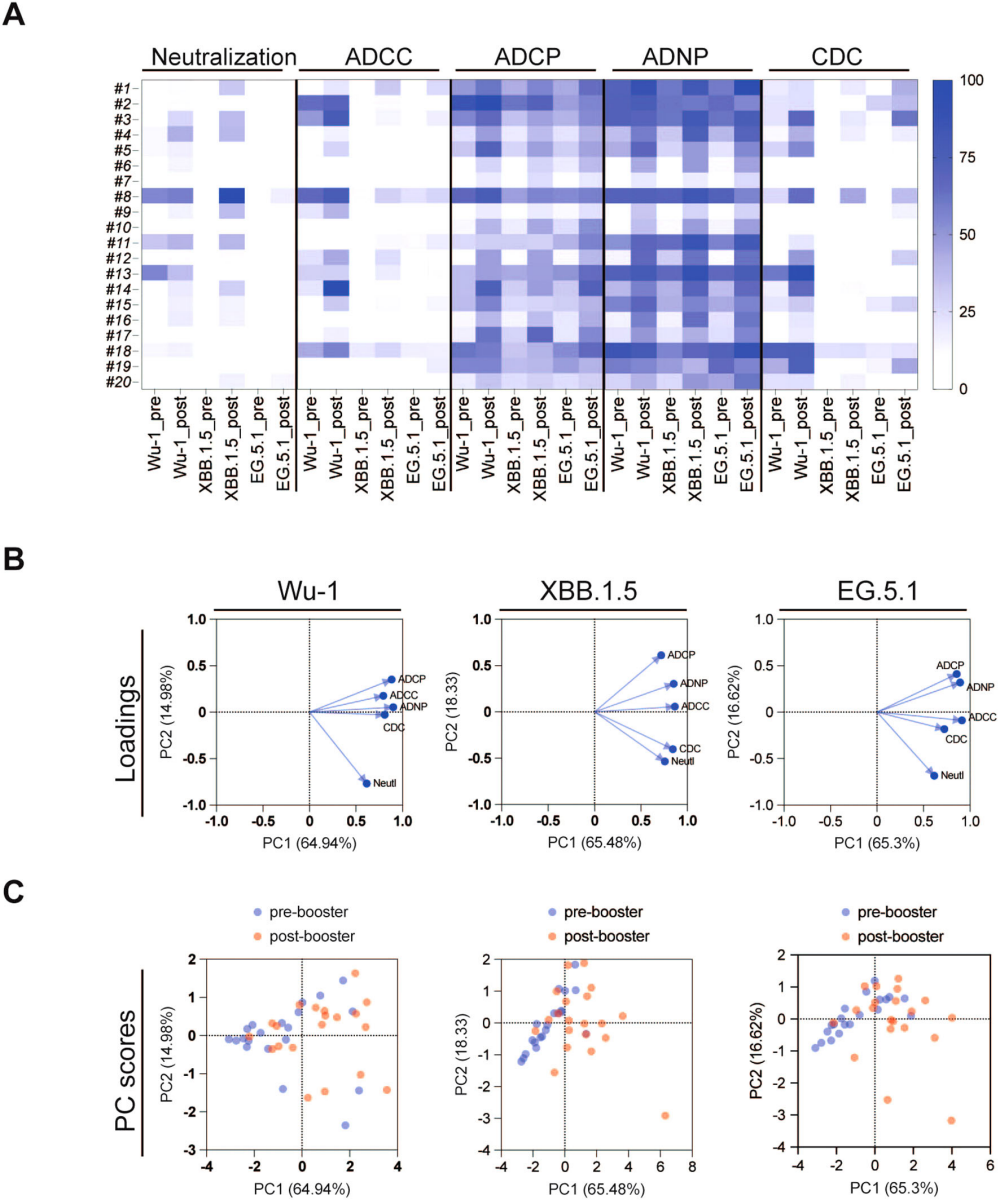
**(A)** Heatmap analysis of pseudovirus neutralizing antibody and Fc effector functional antibody responses against SARS-CoV-2 variants Wu-1, XBB.1.5, and EG.5 pre- and post-COVID-19 XBB.1.5 booster vaccination. Data was normalized in each assay by defining the smallest value as equal to 0% and the largest value equal to 100%. **(B, C)** Principal component analysis (PCA) was conducted on pseudovirus neutralization antibody and Fc effector functional antibody responses pre- and post-COVID-19 XBB.1.5 booster vaccination. **(B)** highlights the variables contributing to the spread of the point. **(C)** shows individual plot with the pre-booster serum in blue and the post-booster serum in red.

We then performed principal component analysis (PCA) to characterize composite antibody profiles pre- and post-COVID-19 XBB.1.5 vaccination. PCA is a multi-dimensional analysis technique that helps simplify and visualize complex data. PCA demonstrated the clustering of antibody responses pre- and post-vaccination as indicated in **Figure 5**. For the Wu-1 variant, a distinct phenotype was observed between neutralization antibodies and Fc-effector antibodies. The distinct clustering suggested that neutralizing and Fc-effector antibodies differed substantially to Wu-1 spike.

In contrast, the PCA analysis of responses against the more recent variants XBB.1.5 and EG.5 revealed notable changes in antibody profiles, with the neutralization and CDC and ADCC Fc-effector antibody profiles aligning more closely than with the Wu-1 strain. The PC score plot (**Figure 5**) further illustrates the distinctions between the pre- and post-COVID-19 XBB.1.5 vaccination groups across all three variants. The difference between these groups were particularly pronounced, demonstrating that the booster vaccine elicited a robust immune response. These findings underscore the critical role of booster immunizations in enhancing antibody responses, not only for neutralization but also for Fc-mediated processes ADCC, CDC, ADCP, and ADNP.

## 3 Discussion

We developed a panel of functional Fc-effector assays to measure ADCC, CDC, ADCP, and ADNP against SARS-CoV-2 spike variants. The ADCC and CDC assays built upon our prior experience generating target cell lines with inducible expression of the variant spikes, along with luciferase and GFP reporters. The ADCP and ADNP assays incorporated a novel approach of fluorescently labeling variant spike VLPs with DiD dye and measuring phagocytosis by flow cytometry. The assay methods were optimized using reference sera from BEI Resources along with SARS-CoV-2 positive and negative (pre-pandemic) controls. Results were highly reproducible for each assay type. These assays are advantageous in that they measure true effector functions in terms of cytotoxicity of target cells and phagocytosis of target antigen. The ADCC, ADCP, and ADNP assays also use immortalized cell lines as the effector cells, which reduces variability compared to primary blood mononuclear cells (PBMCs). These assays may better correspond to *in vivo* responses than reporter-based mechanism-of-action (MoA) assays.

We then measured the pseudovirus neutralizing and Fc effector antibody profiles of 20 healthy adults pre- and post-COVID-19 XBB.1.5 booster vaccination using our panel of assays. Prior to vaccination, cross-neutralizing antibodies against XBB.1.5 and EG.5 variants were minimally detectable, while cross-functional Fc-effector antibodies were present at higher baseline levels. The COVID-19 XBB.1.5 booster vaccination significantly boosted both neutralizing and Fc-effector antibodies in magnitude and breadth. The greatest increase in neutralizing antibodies was against the XBB.1.5 strain, while Fc-effector functional antibodies had similar fold-increases in antibody titers against the breadth of SARS-CoV-2 variants tested. Neutralizing and Fc-effector antibodies were most highly correlated at baseline (prior to booster vaccination) but were less correlated post-vaccination, reflecting differential boosting of neutralizing vs Fc-effector antibodies by the monovalent vaccine (18, 27). Our population had previous exposures to the SARS-CoV-2 spike protein, both in the forms of vaccination and natural infection. Participants’ prior responses to the SARS-Cov-2 spike protein likely influenced their antibody responses to the COVID-19 XBB.1.5 booster vaccination, which included a mixture of anamnestic responses to Wu-1 epitopes and primary responses to XBB.1.5.

While neutralizing antibodies are known to correlate with protection against COVID-19, the role of Fc-effector antibodies is incompletely understood. However, increasing evidence has found that Fc-effector activity plays a key role in antibody-mediated protection. Mackin, et al. found that Fc-γR interaction is necessary for vaccine-mediated protection against SARS-CoV-2 variants in a mouse model (28). Gorman, et al. further demonstrated that both neutralizing and Fc-effector functions contribute to protection of non-human primates (5). Winkler et al. additionally found that SARS-CoV-2 neutralizing antibodies require intact Fc-effector functions for optimal protection, although not for prophylaxis (29). Additionally, multiple studies have shown that non-neutralizing antibodies can provide protection via Fc-effector functions (1, 30). This is important because SARS-CoV-2 variants that evade neutralizing antibodies may remain susceptible to non-neutralizing antibodies that elicit Fc-effector functions. For example, a recent study by Paciello et al. found that Wu-1 neutralizing antibodies that lost neutralizing activity to SARS-CoV-2 Omicron variants retained Fc-effector activity. Furthermore, the neutralizing antibodies with the most potent Fc effector activity bound to the spike N-terminal domain, followed by class 3 epitopes in the spike receptor binding domain (RBD) (31). In contrast, antibodies that bound to the class 1/2 epitopes of the RBD with most potent neutralizing activity had the lowest Fc-effector activity. Interestingly, Lee et al. found that Fc-effector antibodies persist longer than neutralizing antibodies following mild to moderate COVID-19 in humans (32). Taken together, these studies indicate that Fc-effector antibodies play an important role in protection against COVID-19, particularly when neutralizing antibodies wane or escape variants emerge.

This study had some limitations. In terms of assay development, the surface expression of spike variants on the ADCC and CDC target cell lines was highly similar but not standardized, nor was the amount of VLP produced from the cell lines. ADNP and ADCP were assayed at only one serum concentration due to limitations of the assay throughput, and this concentration may not have elicited the peak phagocytosis for a given serum. While effector cell HL-60 cells were differentiated down a neutrophil pathway for ADNP, they do not represent fully mature neutrophils and thus only serve as a model. To perform analogous assays for other pathogens would require generation of new target cell lines with inducible expression of the immunogens of interest, in addition to cell lines with inducible production of VLPs. There is inherent variability in this process, which limits standardization across pathogens. There is also inherent delay in the development and production of new cell lines, which limits timeliness of response. In terms of our clinical cohort, the sample size was small and the cohort was predominantly white race and non-Hispanic ethnicity enrolled at a single center, which may limit generalizability of findings. Participants had diverse prior exposures to SARS-CoV-2 infections and vaccines, and both Moderna and Pfizer XBB.1.5 monovalent booster were administered, reflecting the complexity of real-world scenarios in assessing responses to booster vaccinations. The assays measured Fc-effector antibodies against three SARS-CoV-2 strains (Wu-1, XBB.1.5, and EG.5.1) following the COVID-19 XBB.1.5 monovalent booster vaccination, but not the more recent variant-specific vaccines that account for new changes in the virus spike protein. Importantly, ADCC, CDC, ADCP, and ADNP are all Fc-mediated pathways, but are mediated by different cells, Fc receptors, and immunoglobulin isotypes *in vivo*, and not all of these factors could be accounted for in the *in vitro* assays. The durability of immunity and other components of the immune system (e.g. T cells) were not assessed. Future research aimed at standardizing Fc effector assays, increasing throughput, and recapitulating *in vivo* functions might facilitate a better understanding of the role of these antibodies across immunogens and pathogens.

In conclusion, we generated a panel of functional Fc effector antibody assays for SARS-CoV-2 variants and characterized the antibody profile of healthy adults pre- and post-COVID-19 XBB.1.5 monovalent booster vaccination. Overall, our study demonstrated that booster vaccination significantly enhanced the magnitude, quality, and breadth of antibody responses against SARS-CoV-2 variants. Understanding the full range of antibody-mediated protection could improve the development and optimization of future therapeutics and vaccines.

## 4 Methods

### 4.1 Study participants and serum samples

Twenty healthy adults who were receiving the XBB.1.5 monovalent COVID-19 vaccine per standard-of-care were recruited for blood collection pre- and 1-month post-vaccination at Emory Children’s Center- Vaccine Research Clinic (IRB00022371). Demographic and clinical data, including self-reported history of COVID-19 diagnoses or vaccines, were collected on case report forms and entered a secure, HIPAA-compliant REDCap database. For the development of assays, biobanked sera collected from twelve healthy adults enrolled into a phlebotomy protocol prior to onset of the COVID-19 pandemic were utilized as negative controls (IRB00045690). All study procedures were performed following written informed consent. Both protocols utilized in this study were approved by the Institutional Review Board at Emory University. All serum samples underwent complement inactivation by heating at 56°C for 45 min prior to analysis. Serum samples were stored at −80°C until use.

### 4.2 Production of SARS-CoV-2 pseudovirus and pseudovirus neutralization assay

SARS-CoV-2 spike pseudovirus production was previously described (22–24). To construct SARS-CoV-2 pseudoviruses, a recombinant plasmid containing the spike protein gene from SARS-CoV-2 variant of ancestral (Wu-1), Omicron XBB.1.5 or EG.5 was co-transfected with an HIV-1 lentiviral backbone plasmid (pNL4.3.Luc.R-E-) into 293T cells, and then harvested from the supernatant. Pseudoviruses were filtered (0.45-µm pore size) and stored at −80°C until use. Pseudoviruses were titrated using a luminescence read-out on a TopCount^®^ NXTTM luminometer (Packard Instrument Company; Meriden, CT, USA). Pseudovirus-neutralization assays were performed as previously described (22, 24). Briefly, 293T-ACE2 cells were cultured in DMEM with 10% FBS and 200 µg/mL hygromycin, seeded into 96-well plates, and incubated overnight at 37°C. Heat-inactivated serum was diluted 3-fold starting at 1:30 in 5% FBS DMEM in 96 well plates. Each well was mixed with equal volume of SARS-CoV-2 pseudovirus (3 × 10^4^ RLU/mL) and incubated for 1 hour. After removing the media, 100 µL of the mixture was added to the cells. Controls included uninfected cells (cell control) and cells with pseudovirus only (virus control). After 48 hours at 37°C, luminescence was measured using Britelite™ Plus luciferase substrate. Neutralization curves were generated by 4-point non-linear regression, with titers expressed as IC50. The lower limit of detection (LLOD) was 1:30 with undetectable titers assigned a value of ½ LLOD.

### 4.3 Development of ADCC and CDC assays

A reporter-only cell line with dual expression of luciferase and EGFP reporters was generated previously (25) using T-REx-293 cells (Invitrogen, Carlsbad CA) expressing the tetracycline repressor protein (TetR). This cell line was used as the control cell line and for downstream cell line development. The target cell line with tetracycline-inducible expression of Wu-1 SARS-CoV-2 spike was also generated previously (26). XBB.1.5 and EG.5 target cell lines were generated using the same approach as the Wu-1 target cell line. Briefly, the reporter-only cells were transfected with pcDNA5/TO (p) CoV2_S_XBB.1.5 or pcDNA5/TO (p) CoV2_S_EG.5. Following puromycin selection, single clones were expanded and characterized by flow cytometry using serum from SARS-Cov-2 vaccine immunized serum (BEI cat # NRH 28557) (**Figure 1B**). After characterization, the individual clone with the highest surface expression of spike protein was amplified and named the “SARS CoV-2 S_XBB.1.5” and “SARS CoV-2 S_EG.5”. Western blot was performed to confirm the cellular expression of spike proteins. These cell lines were used for subsequent experiments.

Development of the SARS-CoV-2 spike ADCC assay was previously described (26). Briefly, the serum samples were serially diluted in duplicate in 96-well V-bottom plates with AIM V™ (Gibco 12055091) medium starting at 1:30 with 3-fold dilutions. The effector: target (E: T) cell ratio of 2:1 and 4 h incubation time were the optimal experimental condition. After addition of Britelite Plus luciferase reporter reagent (PerkinElmer) the plate was incubated for 5 min and Relative Luminescence Units (RLU) was read on a luminometer (TopCount NXT Luminescence Counter).

To develop the CDC assay, the target cell line was cultured in DMEM medium with 10% FBS supplemented with 200 µl/mL of Zeocin, 5 µl/mL of Blasticidin and µl/mL of Puromycin. Serum samples were serially diluted in the AIM V medium in 96 well U-bottom plate and transferred 25 µL of diluted serum into 96-well clear bottom black plates. Accutase was used to detach the cells that were cultured and induced with doxycycline in a 75cm^2^ flask 18-24 hours before the assay. Then, 50 µL of 4x10^4^ cells/well were added to the plates and incubated for 20 minutes at 37°C. Subsequently, 25 µL/well of optimized Guinea pig complement (1:20 diluted in AIM-V) was added. Plates were incubated for 20-28 hours at 37°C. Then, 100 µL of ambient temperature Britelite Plus luciferase substrate was added to each well and mixed thoroughly using multiple pipettor. The bottom of the plate was covered with Black Vinyl sealing tape (Thermo Scientific: 236703) and the top of the plate was covered with Adhesive Clear Polyester Seal Film (GE Healthcare: 7704-0001). The plate was incubated for 2 min and the luminescence (RLU) was measured in a luciferase plate reader (TopCount NXT Luminescence Counter). The complement concentration and incubation time were selected to minimize spontaneous target cell killing and maximize signal-to-noise ratio for optimal experimental conditions. The percentage of cytotoxicity was calculated based on the luminescence readings using the following formula:


$$CDC\;cytotoxicity\left(\%\right)=\frac{RLU*\left(no\;antibody\right)-RLU\left(with\;antibody\right)}{RLU\left(no\;antibody\right)}x100$$

### 4.4 Production and staining of virus-like particles

To develop a cell line to inducibly produce SARS-CoV-2 spike virus-like particles, T-Rex 293 cells were transfected with the pCDNA4/TO plasmid harboring the HIV gag gene. Stable HIV Gag-expressing clones were selected using Zeocin at a final concentration of 200 µg/mL. Individual colonies were picked using a pipette tip and transferred to separate wells of a 12-well plate containing DMEM with 10% tetracycline-free FBS, 1% penicillin-streptomycin, and 200 µg/mL Zeocin. As the cells grew, they were expanded by transferring them to larger culture dishes. The expression of the Gag protein was characterized by Western blotting. The clone with high Gag expression and low background was expanded and named the Gag#46 cell line. The Gag#46 cell line will be used both as a Gag/S VLP production cell line and as a negative control for Gag-only VLP production. Using the same strategy, the pCDNA5/TO (puro) plasmid harboring the spike (S) gene from SARS-CoV-2 variants was transfected into T-Rex293 cell line. The dual-expressing clones were selected with 200 µg/mL Zeocin and 2 µg/mL Puromycin. Verification of the expression of HIV Gag and SARS-CoV-2 spike proteins was performed by Western blotting and immunofluorescence using specific antibodies against HIV Gag and SARS-CoV-2 spike proteins. This cell line will be used to produce VLPs containing the spike protein upon the addition of doxycycline (DOX).

To stain the VLPs with DiD dye, we first seeded the VLP production cells in 75 cm² flasks one day before the experiment, ensuring that they reached 80% confluency on the day of the experiment. We aspirated the medium and add 8 mL of DMEM without FBS containing 5 µL/mL of DiD dye (1:200 dilution). We incubated the cells at 37°C for 20 minutes. We aspirated the DiD dye medium and added DMEM with 10% FBS containing 1 µg/mL of doxycycline to induce particle production. We then incubated the cells for 40-48 hours to allow for particle production. We harvested the cell culture supernatant containing the produced particles and centrifuged at 2000 x g for 10 minutes to remove cell debris. We filtered the supernatant through a 0.45 µm filter to further remove debris. We layered the filtered supernatant onto 4 mL of a 20% sucrose cushion in a 38 mL ultracentrifuge tube and ultracentrifuged at 100,000 x g for 2 hours to pellet the particles. Finally, we resuspended the purified particle pellet in PBS.

### 4.5 Development of ADCP and ADNP assays

For the ADCP assay, we diluted the test serum samples and control serum samples to the desired concentrations in culture medium in a 96-well U-bottom plate. We incubated the purified particles at an optimized p24 concentration with the diluted serum samples for 20 minutes at 37°C. As controls, we included particles only (no serum). We then added THP-1 effector cells to the serum-particle mixtures at a density of 3×10^5^ cells per well. We incubated the mixture for 18 hours at 37°C in a 5% CO_2_ incubator to allow phagocytosis to occur. After the incubation period, we washed the cells with PBS containing 2% FBS to remove unbound particles. We collected the THP-1 cells by gentle centrifugation and wash them twice with PBS containing 2% FBS. We resuspended the cells in PBS containing 2% FBS for flow cytometry analysis. Gag-only particles were used as controls in parallel with a positive serum and a known negative control serum.

We measured the phagocytosis of DiD-labeled particles by THP-1 cells using flow cytometry. We gated on THP-1 cells and measure the fluorescence intensity of DiD to assess phagocytosis. We analyzed the flow cytometry data to determine the percentage of THP-1 cells that had phagocytosed DiD-labeled particles. We compared the phagocytosis rates between test serum, control serum, and particles-only control to evaluate the specific effect of the test serum on ADCP. We performed statistical analysis to determine the significance of the differences observed.

To perform the ADNP assay, we first differentiated HL-60 cells. To do this, we cultured HL-60 cells in complete RPMI-1640 medium, maintaining them at a density of 2-5 x 10^5^ cells/mL in a T-75 flask, and ensured they remained in the exponential growth phase. To begin differentiation, we adjusted the cell density to 1 x 10^5^ cells/mL in complete RPMI-1640 medium, adding DMSO to a final concentration of 1.3% (v/v). We mixed thoroughly by gently swirling the flask. We incubated the cells in a CO_2_ incubator at 37°C with 5% CO_2_. After 5 days, we harvested the cells by centrifugation at 300 x g for 5 minutes. We washed the cells with PBS to remove any residual DMSO, then resuspended them in complete RPMI-1640 medium without DMSO for downstream applications. We confirmed differentiation by staining with CD11b and analyzing by flow cytometry. The procedure for the ADNP assay was identical to that of the ADCP assay, with the only difference being the use of differentiated HL-60 effector cells instead of THP-1 cells during incubation with serum and differentiated HL-60 cells.

Statistical analysis and graphing were performed in GraphPad Prism version 10. For neutralization analysis, IC_50_ were compared using a Mann–Whitney test of log-transformed values. Nonlinear regression analysis of the raw values was performed for fitting the line. The correlation between the neutralization IC50 value and Fc function values of spike-specific antibodies was analyzed by Pearson correlation coefficients. Comparisons of neutralization and Fc effector function responses between pre-booster and post-booster were performed using the paired T test. Data were presented as geometric means ± geometric standard deviations. Differences were deemed statistically significant with *P < 0.05, **P < 0.01, ***P < 0.001 and ****P < 0.0001. Receiver operating characteristic (ROC) analyses were performed to estimate the cutoff values.

## Data Availability

The raw data supporting the conclusions of this article will be made available by the authors, without undue reservation.
