# Supplementary material for: Fc-effector functional antibody assays for SARS-CoV-2 variants of concern
Source: Front Immunol. 2025 May 20;16:1571835. doi: 10.3389/fimmu.2025.1571835 (PMC12130042; doi:10.3389/fimmu.2025.1571835)
Supplement: Supplementary file 1 [file DataSheet1.docx]

**SARS-CoV-2 naive serum**

**Immunization serum**

**A**

**B**

**C**

**D**

***Supplementary Figure S1.*** Reproducibility of Antibody-dependent cellular phagocytosis (ADCP) assay using (A) SARS-CoV-2-positive BEI reference sera (NRH-28557) and (B) pre-pandemic SARS-CoV-2-naive sera with the same source of THP-1 cells on 2 different days to measure the assay variability. Percentage of cells with phagocytosis measured by flow cytometry is shown on the y-axis and serum concentration is shown on the x-axis. (C) and (D) show the corresponding Pearson correlations between Days 1 and 2 and the p-values.

***Supplementary Figure S2.*** Reproducibility of Antibody-dependent neutrophil phagocytosis (ADNP) assay using (A) SARS-CoV-2-positive BEI reference sera (NRH-28557) and (B) pre-pandemic SARS-CoV-2-naive sera with the same source of 6-day DMSO-differentiated HL60 cells across two different days to measure the assay variability. Percentage of cells with phagocytosis measured by flow cytometry is shown on the y-axis and serum concentration is shown on the x-axis. (C) and (D) show the corresponding Pearson correlations between Days 1 and 2 and the p-values.

Fluorescence Intensity

Control

Wu-1

XBB1.5

EG5.1


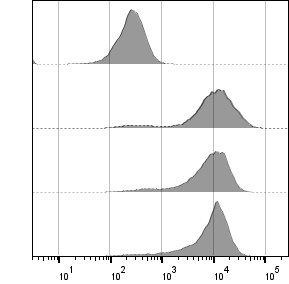


|  |  |  |
| --- | --- | --- |
| **Cell ID** | **%** | **MFI** |
| **Control** | 0.25 | 4897 |
| **Wu-1** | 91 | 11002 |
| **XBB1.5** | 89 | 9187 |
| **EG5.1** | 90 | 9646 |

***Supplementary Figure S3.*** Flow cytometry analysis of the ADCC target cell lines to evaluate the induced surface expression of SARS-CoV-2 spike protein variants Wuhan-1, XBB1.5, and EG5.1 spike proteins, respectively) using BEI reference serum (NRH-28557) as the primary antibody and APC-labeled anti-human antibody as the secondary. MFI, median fluorescence intensity. % represents percentage of positive cells with surface spike expression.

***Supplementary Figure S4.*** Optimization of the complement-dependent cytotoxicity (CDC) assay. The optimal complement concentration (1/40 vs. 1/20 dilution) was determined using XBB1.5 target cells in the presence of four SARS-CoV-2-positive sera (BEI reference sera NRH-28557, NRH-28563, and vaccinated “Donor” sera) and two SARS-CoV-2 naïve “healthy” sera over a 28-hour incubation period. The cytotoxicity, or percentage of target cell death, was calculated based on the luminescence readings of (decrease in relative luminescence units, RLUs).

**Supplementary Figure S5.** Optimization of the complement-dependent cytotoxicity (CDC) assay. The effect of incubation time on CDC activity was assessed. The optimal CDC response of XBB1.5 target cells with guinea pig complement in the present of positive and negative samples at incubation times of 6, 12, and 28 hours was observed.

**Supplementary Figure S6.** Receiver operator curve (ROC) analyses to determine the optimal thresholds for the CDC from 12 individual serum samples collected prior to COVID-19 pandemic and 20 vaccine immunized individuals for working out cutoffs against the Wu-1 and the Omicron variants (XBB1.5 and EG5.1).

**Supplementary Table S1: ROC curve analyses to determine CDC cut-off titer**


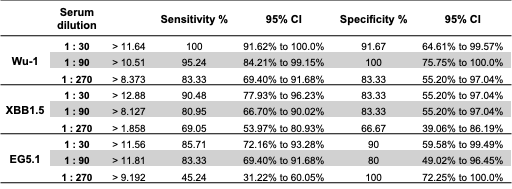

**Supplementary Figure S7.** Reproducibility of Complement-Dependent Cytotoxicity (CDC) Assay using (A) SARS-CoV-2-positive BEI reference sera (NRH-28557) and (B) pre-pandemic SARS-CoV-2-naive sera against EG.5.1 target cells on different days under the same conditions. The percentage of cell death is shown on the y-axis and serum concentration is shown on the x-axis. (C) and (D) show the corresponding Pearson correlations between Days 1 and 2 and the p-values.
